# An engineered 5-helix bundle derived from SARS-CoV-2 S2 pre-binds sarbecoviral spike at both serological- and endosomal-pH to inhibit virus entry

**DOI:** 10.1080/22221751.2022.2095308

**Published:** 2022-08-05

**Authors:** Xi Lin, Liyan Guo, Sheng Lin, Zimin Chen, Fanli Yang, Jing Yang, Lingling Wang, Ao Wen, Yanping Duan, Xindan Zhang, Yushan Dai, Keqing Yin, Xin Yuan, Chongzhang Yu, Bin He, Yu Cao, Haohao Dong, Jian Li, Qi Zhao, Guangwen Lu

**Affiliations:** a West China Hospital Emergency Department (WCHED), State Key Laboratory of Biotherapy, West China Hospital, Sichuan University, Chengdu, People’s Republic of China; b Disaster Medicine Center, West China Hospital, Sichuan University, Chengdu, People’s Republic of China; c Laboratory of Aging Research and Cancer Drug Target, State Key Laboratory of Biotherapy and Cancer Center, National Clinical Research Center for Geriatrics, West China Hospital, Sichuan University, Chengdu, People’s Republic of China; d School of Basic Medical Sciences, Chengdu University, Chengdu, People’s Republic of China; e College of Food and Biological Engineering, Chengdu University, Chengdu, People’s Republic of China

**Keywords:** Broad-spectrum inhibition, 5-helix bundle (5HB), SARS-CoV-2, structural basis, viral entry

## Abstract

Severe acute respiratory syndrome coronavirus 2 (SARS-CoV-2) and related sarbecoviruses enter host cells by receptor-recognition and membrane-fusion. An indispensable step in fusion is the formation of 6-helix bundle by viral spike heptad repeats 1 and 2 (HR1 and HR2). Here, we report the construction of 5-helix bundle (5HB) proteins for virus infection inhibition. The optimal construct inhibits SARS-CoV-2 pseudovirus entry with sub-micromolar IC50. Unlike HR2-based peptides that cannot bind spike in the pre-fusion conformation, 5HB features with the capability of binding to pre-fusion spike. Furthermore, 5HB binds viral HR2 at both serological- and endosomal-pH, highlighting its entry-inhibition capacity when SARS-CoV-2 enters via either cell membrane fusion or endosomal route. Finally, we show that 5HB could neutralize S-mediated entry of the predominant SARS-CoV-2 variants and a wide spectrum of sarbecoviruses. These data provide proof-of-concept evidence that 5HB might be developed for the prevention and treatment of SARS-CoV-2 and other emerging sarbecovirus infections.

## Introduction

The ongoing global pandemic of coronavirus disease 2019 (COVID-19), caused by severe acute respiratory syndrome coronavirus 2 (SARS-CoV-2), has resulted in 515,748,861 confirmed cases including 6,255,835 deaths as of 10 May 2022 (https://covid19.who.int/). The coronavirus spike (S) glycoprotein is responsible for viral entry into host cells. S comprises two functional subunits, S1 and S2, mediating receptor recognition and membrane fusion, respectively [1,2]. S1 subunit includes a receptor-binding domain (RBD) that recognizes the host receptor of angiotensin-converting enzyme 2 (ACE2)[3,4]. RBD is a crucial target for neutralization antibodies (nAbs) and a key component for vaccine design [5]. While researches and applications of SARS-CoV-2 nAbs and vaccines are developing with an unprecedented speed and have achieved great success, the genetic drift of coronavirus has caused the rapid emergence of viral variants [6,7,8]. To date, the well-recognized and threatening variants of concern (VOC) contain B.1.17 (Alpha), B.1.351 (Beta), B.1.617.2 (Delta), and B.1.1.529 (Omicron), with the characteristics of higher infectivity, antibody resistance, and vaccine breakthrough compared to the prototype coronavirus [9,10,11,12,13,14]. Notably, Omicron is rapidly becoming the dominant pandemic variant, which severely compromises the neutralizing effect of antibodies and plasma from COVID-19 convalescent or vaccinated individuals [9,15]. Thus, exploring broad-spectrum vaccines or drugs to control the emerging variants is highly imperative.

Sarbecovirus contains SARS-CoV-related lineage and SARS-CoV-2-related lineage. Animal-derived coronaviruses isolated from bats, pangolins, and palm civets that share many similarities with SARS-CoV or SARS-CoV-2 are raising concerns for zoonotic transmissions and potential pandemic [16,17,18]. Considering that most SARS-CoV or SARS-CoV-2 specific monoclonal antibodies exert narrow binding efficiency and neutralization breadth [19,20], it is therefore also an urgent issue to explore broadly neutralizing vaccines or drugs to prevent future zoonotic spillovers among other sarbecoviruses.

Due to selective evolution pressure, the amino acid changes in SARS-CoV-2 variants and the sequence differences among sarbecoviruses are more profoundly condensed in the S1 subunit. In comparison, the sequence of S2 fusion machinery is relatively conserved during viral evolution [21,22]. S2 subunit includes heptad repeats 1 and 2 (HR1 and HR2), which mediates viral cellular membrane fusion by forming a 6-helix bundle (6HB). During membrane fusion, S protein undergoes three major structural transformations: in the pre-fusion state, S1 wraps S2 subunit and HR1 is buried by other components [2,23]; in the fusion intermediate state, RBD binding with ACE2 receptor induces the dissociation of S1 and the large conformational change of S2, including fusion peptide insertion into the host cell membrane and the exposure of HR1; finally in the post-fusion state, three HR1 fold into a central trimer core structure and three HR2 bind in the inter-HR1 groove in an antiparallel manner to form the long 6HB fusion core, leading to viral and cellular membrane fusion [2,24,25]. The formation of the 6HB fusion core is a key step in mediating viral entry. Reciprocally, targeting the 6HB fusion core is a prospective strategy to develop broad-spectrum inhibitors. As reported, HR2-derived peptides CP-1, MERS-HR2P, and 2019-nCoV-HR2P interact with the HR1 domain and prevent cellular membrane fusion with SARS-CoV, Middle East Respiratory Syndrome Coronavirus (MERS-CoV), and SARS-CoV-2, respectively [26,27,28]. In addition, EK1 modified from OC43-HR2P showed broad-spectrum inhibition activity against human coronaviruses [28,29]. Besides, antibodies targeting the conserved epitopes in the stem helix of S2 fusion machinery could also sterically disturb S2 structural changes, leading to membrane fusion disruption. These antibodies show broad neutralization activity against beta-coronaviruses [30,31]. Taken together, the S2 subunit, as a relatively conserved site, is an ideal target for vaccine design and antiviral drug development against sarbecoviral transmission.

Here, we screened a series of 5-helix bundle (5HB) proteins and found an optimal 5HB construct (5HB-H2) with broad-spectrum membrane-fusion inhibition activity. 5HB-H2 could actively block the syncytium formation mediated by SARS-CoV-2 S and inhibit the entry of SARS-CoV-2 pseudovirus. In addition, 5HB-H2 was able to bind viral spike at the pre-fusion state as detected by flow cytometry. Notably, 5HB-H2 also bound HR2 peptide (HR2P) in an acidification-insensitive manner, indicating its capacity in entry-inhibition, especially when virus enters via the endosomal route. In particular, the HR2 sequences of the prevalent SARS-CoV-2 variants and concerned sarbecoviruses are highly conserved, suggesting its feasibility to be developed as a universal prevention and treatment strategy against sarbecoviruses. Consistently, 5HB-H2 indeed markedly inhibited viral infection of SARS-CoV-2 variants and other representative sarbecoviruses. Furthermore, the crystal structure of the 5HB-H2/HR2P complex revealed conserved hydrogen bond and hydrophobic interactions mediating S-binding by 5HB and elucidated the basis for the broad-spectrum viral entry-inhibition activity of 5HB. These results suggested that 5HB represented a promising candidate for further drug development in order to controll the ongoing COVID-19 pandemic and the potential cross-species transmission of sarbecoviruses.

## Results

### Fusion-core-guided engineering towards stable 5HB proteins

**Figure 1.**
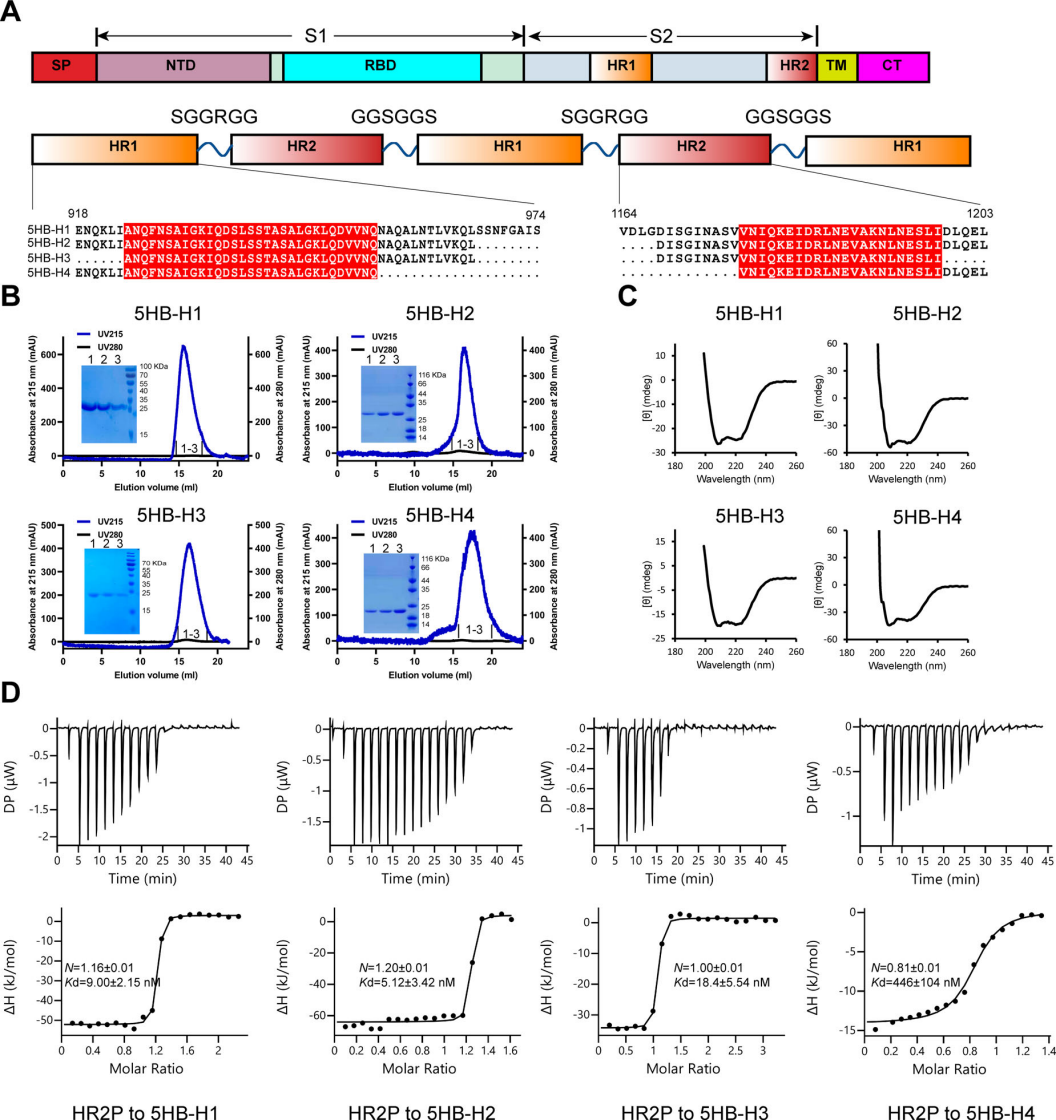
In vitro characterization of the binding between the 5HB proteins and HR2P. (A) Schematic representation of the protein-engineering strategy used to yield the 5HB proteins. SP, signal peptide; NTD, N-terminal domain; RBD, receptor-binding domain; HR1 (heptad repeat 1), HR2 (heptad repeat 2); TM, transmembrane domain; CT, cytoplasmic tail. (B) Solution behaviours of 5HB proteins, including 5HB-H1, 5HB-H2, 5HB-H3 and 5HB-H4, on a Superdex 200 increase 10/300 column. The UV absorbance curves were recorded at 215-nm due to the lack of aromatic residues in 5HB proteins. The inset figure shows the SDS-PAGE analyses of the pooled samples. (C) CD spectra of the 5HB proteins to show their helical structures. (D) Binding of HR2P to the indicated 5HB proteins characterized by ITC.

Guided by the studies on the successful construction of 5HB proteins targeting HIV and MERS-CoV [32,33], we therefore focused on the conserved HR2 region of SARS-CoV-2, aiming to obtain the HR2-responsive 5HB proteins. Based on the SARS-CoV-2 6HB fusion core structure (PDB code: 6LXT)[34], we designed a series of 5HB constructs, which consist of three copies of HR1 and two copies of HR2 alternatively linked by the SGGRGG and GGSGGS linkers, respectively (Figure 1(A)). The longest construct, namely 5HB-H1, spans residues 918-974 of HR1 and residues 1164-1203 of HR2. The other three constructs are truncated ones, including 5HB-H2 with amino acids of HR1 E918-L966 and HR2 D1168-L1203, 5HB-H3 with amino acids of HR1 A924-L966 and HR2 D1168-I1198 and 5HB-H4 with amino acids of HR1 E918-Q954 and HR2 V1176-L1203 (Figure 1(A)). These truncated constructs represent a truncation of several HR1 helical turns and the corresponding HR2 loops either on one side or from both sides (Fig. S1A).

The homogeneous 5HB proteins were subsequently prepared and all featured with typical α helical structure as detected by Circular Dichroism (CD) (Figure 1(B,C)), indicating that all 5HB proteins were properly folded. To evaluate the capacity of 5HB binding to its target of HR2, we initially synthesized a 36-mer peptide derived from HR2 (amino acids: D1168-L1203, hereafter designated as HR2P) and used it for an enzyme-linked immunosorbent assay (ELISA) (Fig. S1B). All 5HB proteins were observed to bind to HR2P, although 5HB-H4 showed less binding efficiency compared with the others. We further titrated HR2P to the individual 5HB proteins (5HB-H1, 5HB-H2, 5HB-H3 and 5HB-H4) by isothermal titration calorimetry (ITC) for a quantitative binding evaluation. The binding affinities were determined to be 9.00 ± 2.15 nM for 5HB-H1, 5.12 ± 3.42 nM for 5HB-H2, 18.4 ± 5.54 nM for 5HB-H3 and 446 ± 104 nM for 5HB-H4, respectively (Figure 1(D)). The result echoes our ELISA observations such that 5HB-H1, 5HB-H2 and 5HB-H3 interact effectively with HR2P but 5HB-H4 binds HR2P with apparently lower efficiency.

### Identification of a 5HB protein with optimal entry-inhibition activity against SARS-CoV-2

**Figure 2.**
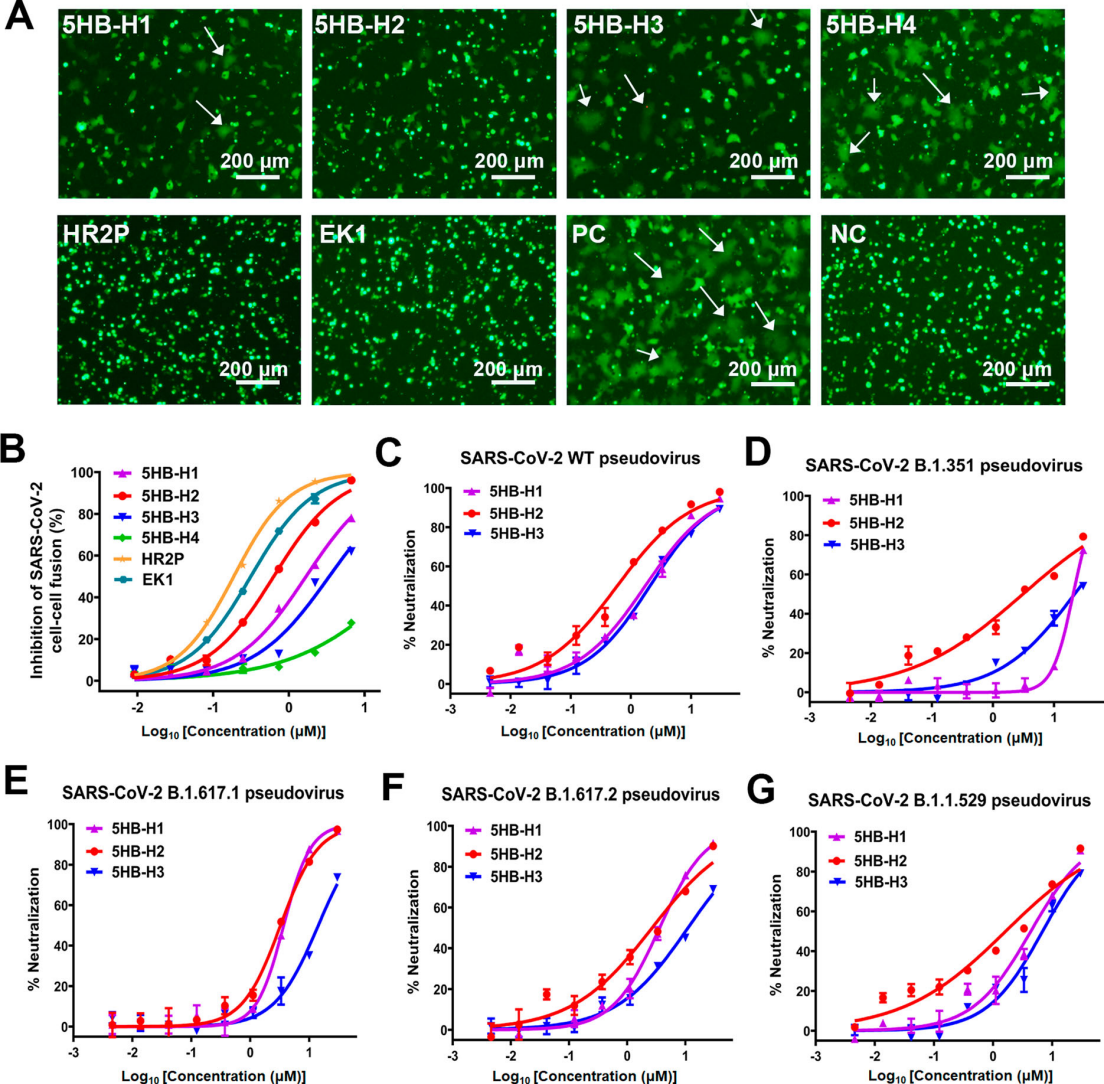
The entry-inhibition activity of the 5HB proteins. (A) Representative images of the S-mediated syncytia formation in the presence of the indicated 5HB proteins (5HB-H1, 5HB-H2, 5HB-H3 and 5HB-H4) or peptides (HR2P and EK1) at the concentration of 10 μM. PC, positive control, 293T/EGFP/S cells with 293T-hACE2 cells. NC, negative control, only 293T/EGFP/S cells. White arrows indicated the fused syncytia. Scale bar: 200 μm. (B) Inhibitory activity of 5HB proteins and peptides on SARS-CoV-2 S-mediated cell-cell fusion. Data are means ± SD of triplicate samples. (C-G) Inhibitory activity of the 5HB proteins (5HB-H1, 5HB-H2, and 5HB-H3) in the pseudovirus infection assays against SARS-CoV-2 of both the prototype (C) and the variant viruses, including B.1.351 (D), B.1.617.1 (E), B.1.617.2 (F), and B.1.1.529 (G), respectively. Inhibition of viral entry was measured according to the reduction in luciferase activities. Data are expressed as means ± SD.

We next evaluated the fusion-inhibition activities of our 5HB proteins, aiming to identify the optimal construct with the most potent efficacy. The initial screening was performed with a cell–cell fusion assay in the presence of 10 μM 5HB proteins or 10 μM HR2P and EK1 peptides. As expected, both HR2P and EK1 could completely block the S-mediated membrane fusion. The complete inhibition of syncytium formation was also observed for 5HB-H1 and 5HB-H2, indicating promising fusion-inhibition capacity for the two constructs. Though the syncytia still formed with 10 μM 5HB-H3, clear reduction in the number of syncytia was recorded. The shortest construct of 5HB-H4, however, only slightly inhibited membrane fusion at the concentration of 10 μM (Figure 2(A)). The half-maximal inhibitory concentrations (IC50s) of the 5HB proteins to inhibit the cell–cell fusion were then quantitatively determined. While HR2P and EK1 individually showed an IC50 of 0.19 μM and 0.31 μM, which were consistent with previous reports [28], the IC50 values for the 5HB proteins were determined to be 1.68 μM for 5HB-H1, 0.63 μM for 5HB-H2, 3.41 μM for 5HB-H3 and over 30 μM for 5HB-H4, respectively (Figure 2(A,B) and Table 1A). These results coincide well with our affinity tests which reveal that 5HB-H2 shows the highest binding affinity whereas 5HB-H4 is of the lowest.

**Table 1.** Fusion-inhibitory activity of 5HB proteins.

(A) IC50 values for the indicated 5HB proteins and peptides determined with SARS-CoV-2 S-mediated cell-cell fusion (μM)						
Proteins	5HB-H1	5HB-H2	5HB-H3	5HB-H4	HR2P	EK1
IC50s	1.68	0.63	3.41	>30	0.19	0.31

(B) IC50 values for the indicated 5HB proteins determined with SARS-CoV-2 and its variant pseudoviruses (μM)					
	SARS-CoV-2 pseudovirus				
Proteins	WT/prototype	B.1.351	B.1.1.529	B.1.617.1	B.1.617.2
5HB-H1	1.73	20.55	4.34	3.68	3.59
5HB-H2	0.59	3.08	1.63	3.25	2.66
5HB-H3	2.07	21.59	6.85	14.24	11.04

We further explored the virus entry-inhibition efficacy of our 5HB proteins by using SARS-CoV-2 pseudoviruses. Because 5HB-H4 was apparently less effective, the other three 5HB constructs were subsequently selected for further tests initially using the pseudovirus of the wild-type strain. As expected, clear inhibition of SARS-CoV-2 pseudovirus infection in an apparently dose-dependent manner was recorded (Figure 2(C)). The IC50 values were determined to be 1.73 μM for 5HB-H1, 0.59 μM for 5HB-H2, and 2.07 μM for 5HB-H3, respectively (Table. 1B). We also investigated the virus entry inhibition capacity of the 5HB constructs against several SARS-CoV-2 variants of concern (VOCs), including B.1.351, B.1.617.1, B.1.617.2 and B.1.1.529 which are the most prevalent variants during the past waves of SARS-CoV-2 pandemic. Echoing the highly conserved sequences and structures for the fusion core among viral variants, the 5HB proteins all showed clear dose-dependent inhibitions (Figure 2(D–G)). For all the tested SARS-CoV-2 VOCs, all 5HB proteins showed slightly reduced neutralization efficacy when compared to the prototype virus (Figure 2(C–G) and Table. 1B). This might result from an increase in the viral entry capacity for these variants due to residue mutations (eg. D614G) that would enhance membrane fusion and viral infectivity [35,36]. Consistent with our cell–cell fusion tests, 5HB-H2 outperformed 5HB-H1 and –H3. It inhibited the infection of B.1.351, B.1.617.1, B.1.617.2 and B.1.1.529 pseudoviruses with an IC50 of 3.08 μM, 3.25 μM, 2.66 μM and 1.63 μM, respectively (Figure 2(D–G) and Table. 1B). Taken together, these results highlight that 5HB-H2 possesses the best inhibitory activity against SARS-CoV-2 of both the prototype and the variant viruses.

### 5HB features with the capability of binding to viral spike in the pre-fusion conformation

**Figure 3.**
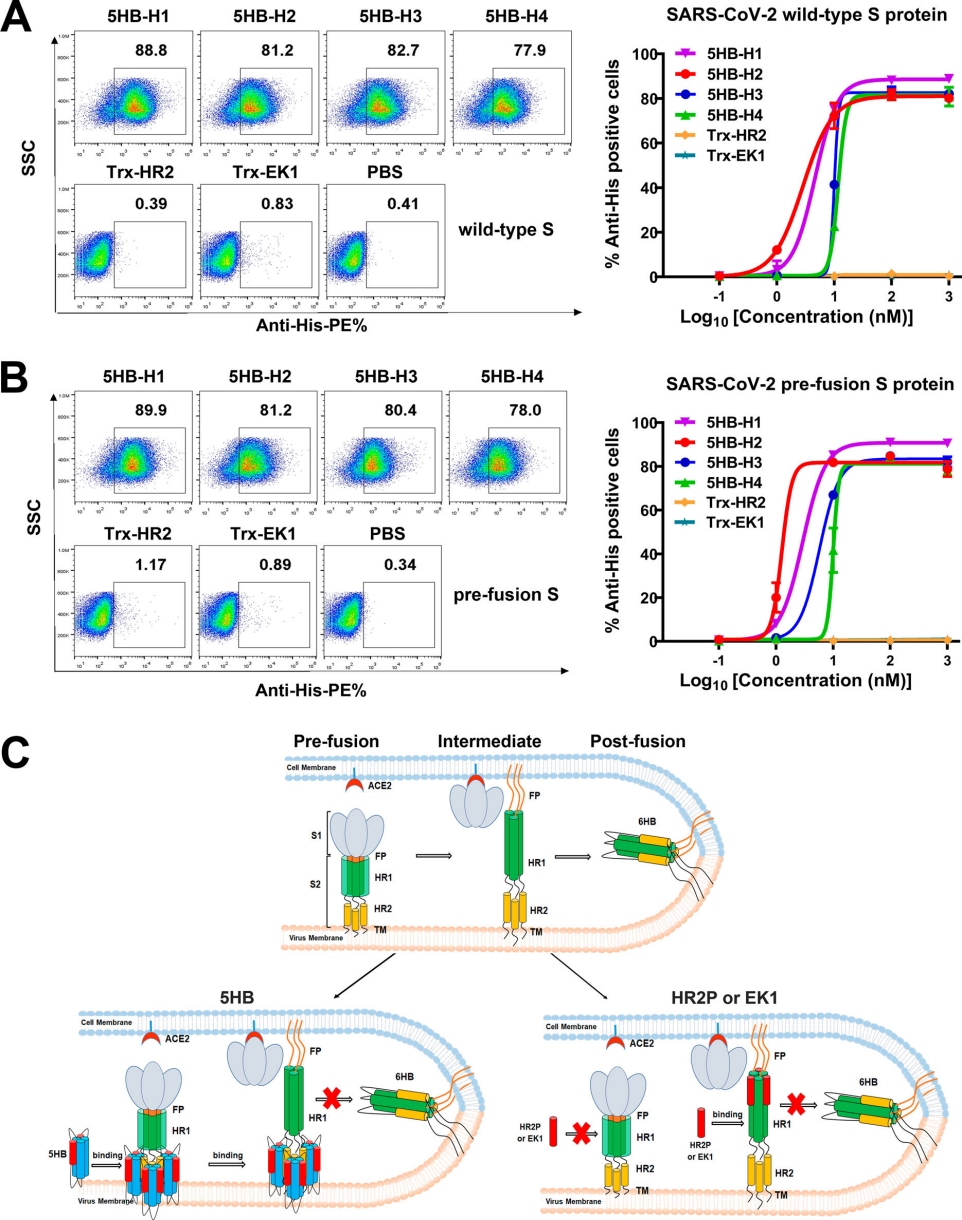
5HB proteins bind both wild-type S and pre-fusion S of SARS-CoV-2. (A-B) Flow-cytometric analysis of binding between SARS-CoV-2 wild-type S (A) or pre-fusion S (B) expressed on the 293T cell surface and 5HB proteins or Trx-HR2 or Trx-EK1 at 1 μM (left). Mean fluorescence intensity was measured with the 10-fold diluted proteins (right). Live cells and single cells were firstly gated. The ratio of stained cells was recorded by flow cytometry. PBS was used as a negative control. (C) A proposed model of SARS-CoV-2 5HB in viral entry inhibition compared with HR2P and EK1. The upper panel shows the process of SARS-CoV-2 spike transition from the pre-fusion conformation to the post-fusion conformation; the lower-left panel highlights the capacity of 5HB to bind pre-S trimer; the lower-right panel shows that HR2P and EK1 peptides cannot bind pre-S trimer. FP (fusion peptide in N-terminal), HR1 (heptad repeat 1), HR2 (heptad repeat 2), TM (transmembrane region).

In the next stage, we further set out to investigate that at which entry step would the 5HB proteins take action to exert inhibition. In the pre-fusion structure of SARS-CoV-2 S trimer, HR1 is buried inside and therefore inaccessible to therapeutics. Accordingly, it is suggested that the HR1-targeting peptides designed based on HR2 theoretically function to bind to HR1 only in the fusion intermediate when HR1 is solvent-exposed but has not fully accommodated HR2 from its own spike. In contrast, the membrane-proximal HR2 domain is proposed to be exposed in pre-fusion S, reminding us that our 5HB proteins should be readily binding to pre-S of SARS-CoV-2. To verify this hypothesis, we expressed SARS-CoV-2 S in 293T cells and tested the binding of individual 5HB proteins with surface-located S by flow cytometry. Expectedly, all 5HB proteins indeed showed a strong binding with clear dose-dependency (Figure 3(A)). We noticed that the wild-type S protein in both the pre-fusion and post-fusion states could be present on the surface of virions or cells, though the pre-fusion forms would commonly dominate over the post-fusion forms prior to receptor binding [1,2]. We therefore further tested the binding of our 5HB proteins with 293T cells expressing SARS-CoV-2 S containing the K986P, V987P, S383C and D985C mutations. These substitutions have been shown to be able to lock the S protein in the pre-fusion state [37]*.* Similar to those observed with wild-type S, all 5HB proteins readily interacted with the pre-fusion-locked S protein on the cell surface (Figure 3(B)), demonstrating that 5HB indeed pre-bound viral spike to block 6HB formation and subsequently inhibited membrane fusion (Figure 3(C)). In comparison to other 5HB constructs, 5HB-H2 showed the best S-binding performance in the flow-cytometric assay (Figure 3(A,B)), echoing our cell–cell fusion and pseudovirus entry inhibition studies which revealed the highest inhibitory activity for this construct.

We also tested the binding of HR1-targeting peptides, including HR2P and EK1, to the wild-type and pre-fusion locked S proteins. Nonetheless, no observable binding was detected for either peptides, even at a concentration of as high as 1 μM (Fig. S2). In light of the low molecular weight and small size of the peptide, it might be difficult to be detected during our flow-cytometric assay. To exclude this possibility, we fused the HR2P and EK1 peptides with an N-terminal Trx tag to obtain a comparable molecular weight with our 5HB proteins and then prepared the resultant proteins (hereafter named Trx-HR2 and Trx-EK1) (Fig. S3A). In the cell–cell fusion assay, both Trx-HR2 and Trx-EK1 showed comparable inhibitory capacity with the HR2P and EK1 peptides, demonstrating that fusion with Trx would not interfere with its fusion-inhibition activity (Fig. S3B-C). Similar to those observed with the peptides, however, no detectable binding was recorded for Trx-HR2 or Trx-EK1 (Figure 3(A,B)). These results indicated that the HR1-targeting peptides, as the membrane fusion inhibitors, could not bind S protein in the pre-fusion state and would only function during the transition state after HR1 was fully exposed (Figure 3(C)). In such circumstances, we believe that our 5HB proteins would have a longer binding window than the HR2P and EK1 peptides.

### 5HB binds to spike in an acidification-insensitive manner

SARS-CoV-2 could enter into host cells either at the plasma membrane under the neutral pH or within the endosomes at low pH [38]. During the endocytosis pathway, the virion would experience several pH decrease events, from the serological pH (∼7.5) to the endosomal pH (∼6.0–5.0) and finally even to the lysosomal pH (∼4.5). It is notable that a lot of protein–protein interactions, as represented by the antigen/antibody binding, could be affected by low pH. The acidic environment of the endosome might therefore interfere with the binding between the viral spike and the potential S-targeting therapeutics, compromising the efficacy when virus enters via the endosomal route. We therefore subsequently investigated the impact of low pH on the binding between 5HB and viral spike.

**Figure 4.**
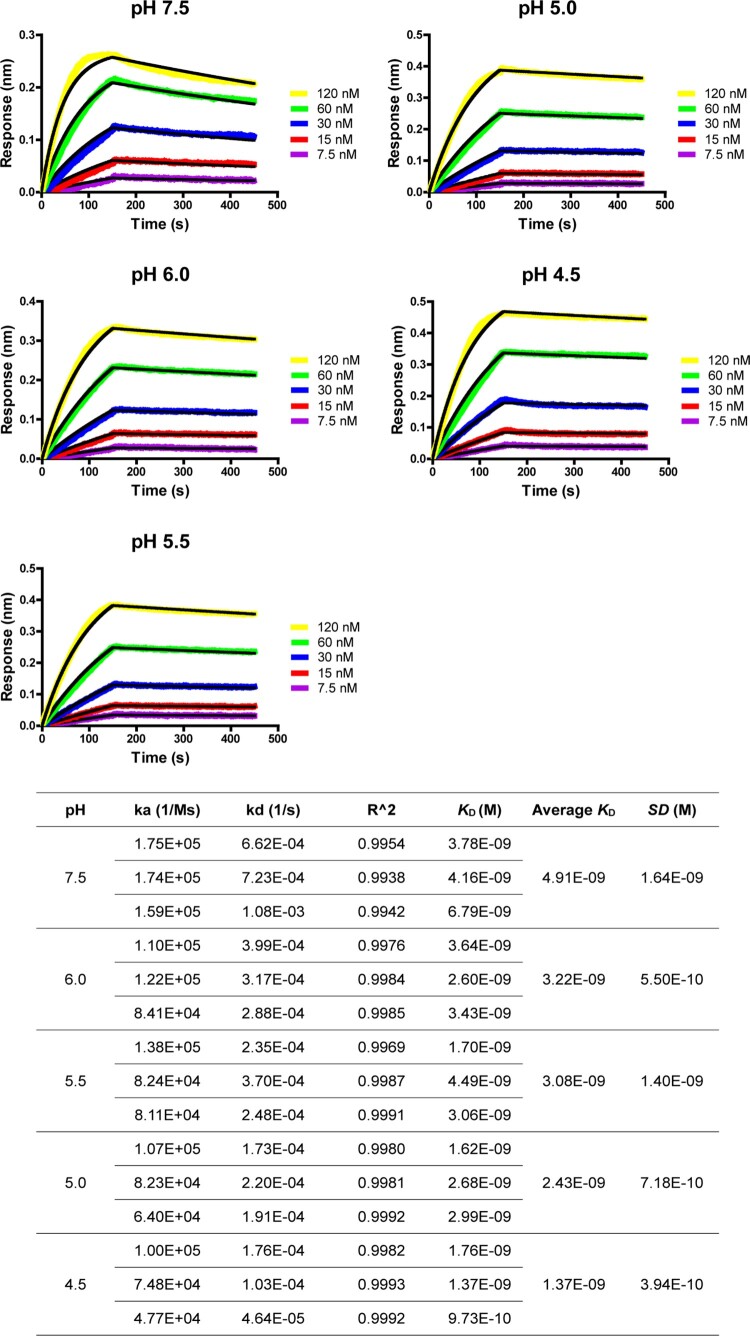
Bio-layer Interferometry sensorgrams and binding affinities of 5HB-H2 with HR2P at pH 7.5, 6.0, 5.5, 5.0 and 4.5, respectively. Three independent experiments are performed and the recorded profiles from one representative experiment are shown. The slow-on/slow-off kinetic data are analyzed by the 1:1 binding model. The calculated kinetic parameters are summarized.

The optimal construct of 5HB-H2 was selected for further exploration and its binding affinity to the HR2P peptide was determined at different pHs, including pH-7.5, −6.0, −5.5, −5.0 and −4.5. Because the HR2P peptide showed decreased solubility in the acidic pH, the ITC test, which required high concentration of the peptide for titration, could not give an accurate measurement at low pH. We therefore resorted to the Bio-layer Interferometry (BLI) experiments for the affinity determination. At pH-7.5, the affinity was determined by BLI to be 4.91 ± 1.64 nM (Figure 4), which is in good accordance with the ITC result. In the parallel BLI tests, intimate binding between 5HB-H2 and HR2P was recorded for all the pH tested. The affinities were determined to be 3.22 ± 0.55 nM, 3.08 ± 1.40 nM, 2.43 ± 0.72 nM and 1.37 ± 0.40 nM at pH-6.0, −5.5, −5.0 and −4.5, respectively (Figure 4). These values highlighted slightly enhanced interactions between 5HB-H2 and HR2P along with the decrease in pH. This observation reveals that 5HB should bind to S in an acidification-insensitive manner, sufficing the 5HB constructs for entry inhibition when SARS-CoV-2 enters via either the plasma membrane fusion or the endosomal routes.

### Structural basis of 5HB binding to SARS-CoV-2 spike

**Figure 5.**
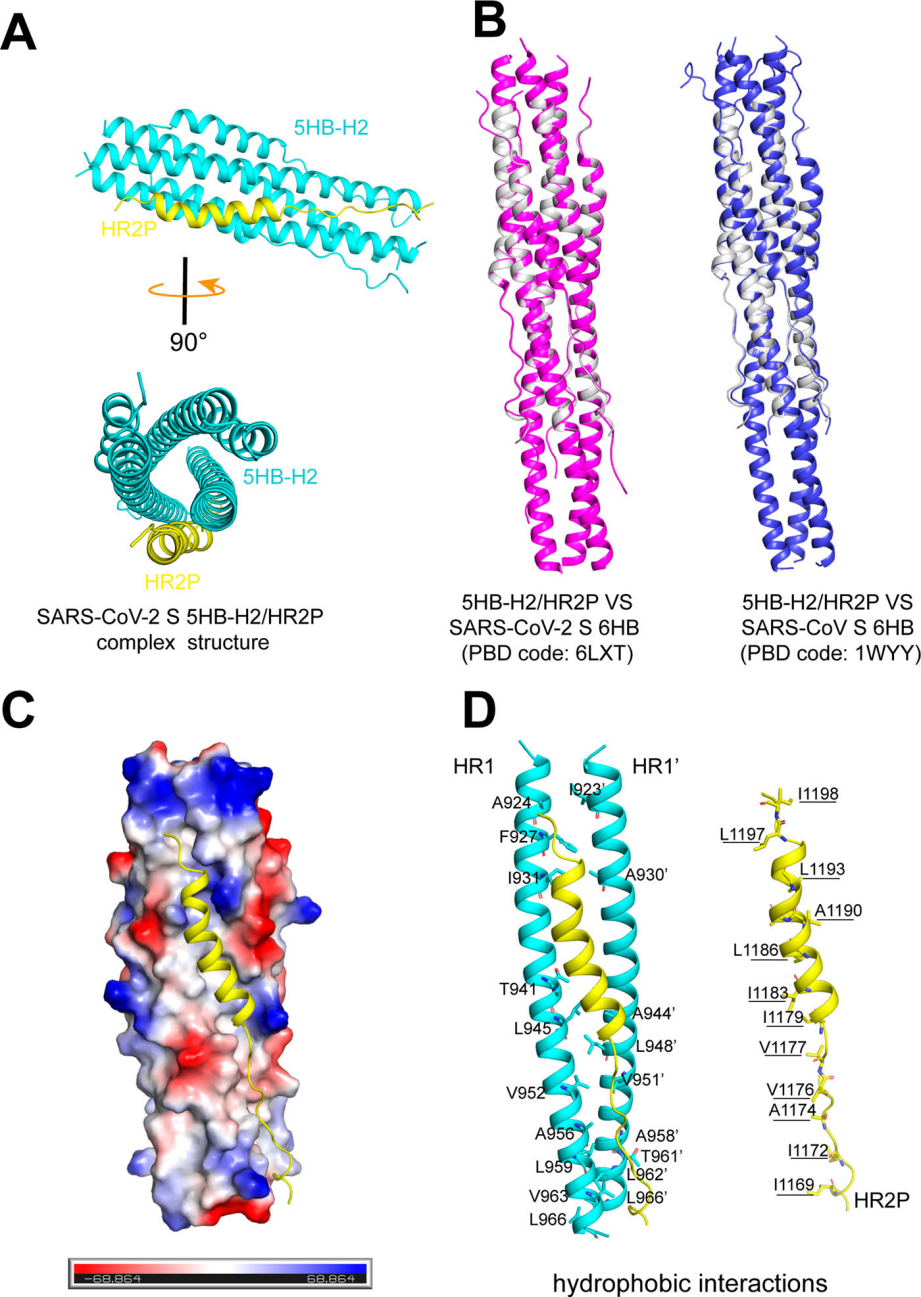
Structure of 5HB-H2/HR2P complex. (A) Overall structure of the 5HB-H2/HR2P complex shown in cartoon. 5HB-H2 are coloured in cyan and HR2P in yellow. Structures in the side view (upper) and top view (lower) are presented. (B) Structural alignments of the 5HB-H2/HR2P complex with SARS-CoV-2 6HB (left) and SARS-CoV 6HB (right). 5HB-H2/HR2P is shown in grey, SARS-CoV-2 6HB in magenta, and SARS-CoV 6HB in blue. (C) The hydrophobic groove between the 5HB-H2 and HR2P. The electrostatic surface of 5HB-H2 is presented. HR2P peptide is shown in cartoon. (D) The detailed hydrophobic interactions between 5HB-H2 and HR2P. Amino acids involved in the hydrophobic contacts are indicated and shown in sticks.

In order to learn the basis of the 5HB/S binding, we further solved the crystal structure of the 5HB-H2/HR2P complex at 1.9 Å resolution. The final structure was refined to *R*_work _= 0.1999 and *R*_free_ = 0.2124, respectively (Table. S1). In the crystallographic asymmetric unit, one 5HB-H2 bound to a single HR2P peptide in a 1:1 binding mode (Figure 5(A)). 5HB-H2 consisted of 5 α-helices and interacted with HR2P to form a coiled-coil complex (Figure 5(A)). The overall structure was similar to the reported structures of SARS-CoV-2 (PDB code: 6LXT) and SARS-CoV (PDB code: 1WYY) 6HBs, showing a root-mean-square deviation (RMSD) of 0.327 and 0.332 Å for the equivalent Cα atoms, respectively (Figure 5(B)). Overall, 5HB-H2 created an extended hydrophobic groove via two of its three central HR1 helices, accommodating HR2P (Figure 5(C)). This groove featured with a series of apolar amino acids from the two adjacent HR1s arranging in an interval way to form a hydrophobic interface. These residues included A924, F927, I931, T941, L945, V952, A956, L959, V963 and L966 in one central helix and I923’, A930’, A944’, L948’, V951’, A958’, T961’, L962’ and L966’ in the other. For HR2P, its helical fold would align I1198, L1197, L1193, A1190, L1186, I1183, I1179, V1177, V1176, A1174, I1172 and I1169 almost on one side of the helix, matching the unoccupied groove in 5HB-H2 for strong hydrophobic interactions (Figure 5(D)). In addition to the hydrophobic contacts, we also observed a series of hydrogen-bond interactions that would contribute to the intimate binding between 5HB-H2 and HR2P. These include 5HB-H2 residues N928, Q935, Q949, N953 and N960 from one central helix and residues Q926’, K933’, D936’, S943’, K947’ and N955’ from the other interacting with the HR2P amino acids A1174, S1175, V1177, I1178, E1182, R1185, A1190, N1192, N1194, E1195, L1197 and I1198 (Fig. S4, Table. S2). These interactive binding details are quite similar to those observed in the 6HB structures, which in turn demonstrates that 5HB-H2 folds properly and binds viral S via canonical HR1/HR2 interactions to inhibit fusion.

### 5HB-H2 shows broad-spectrum inhibition against sarbecoviruses

**Figure 6.**
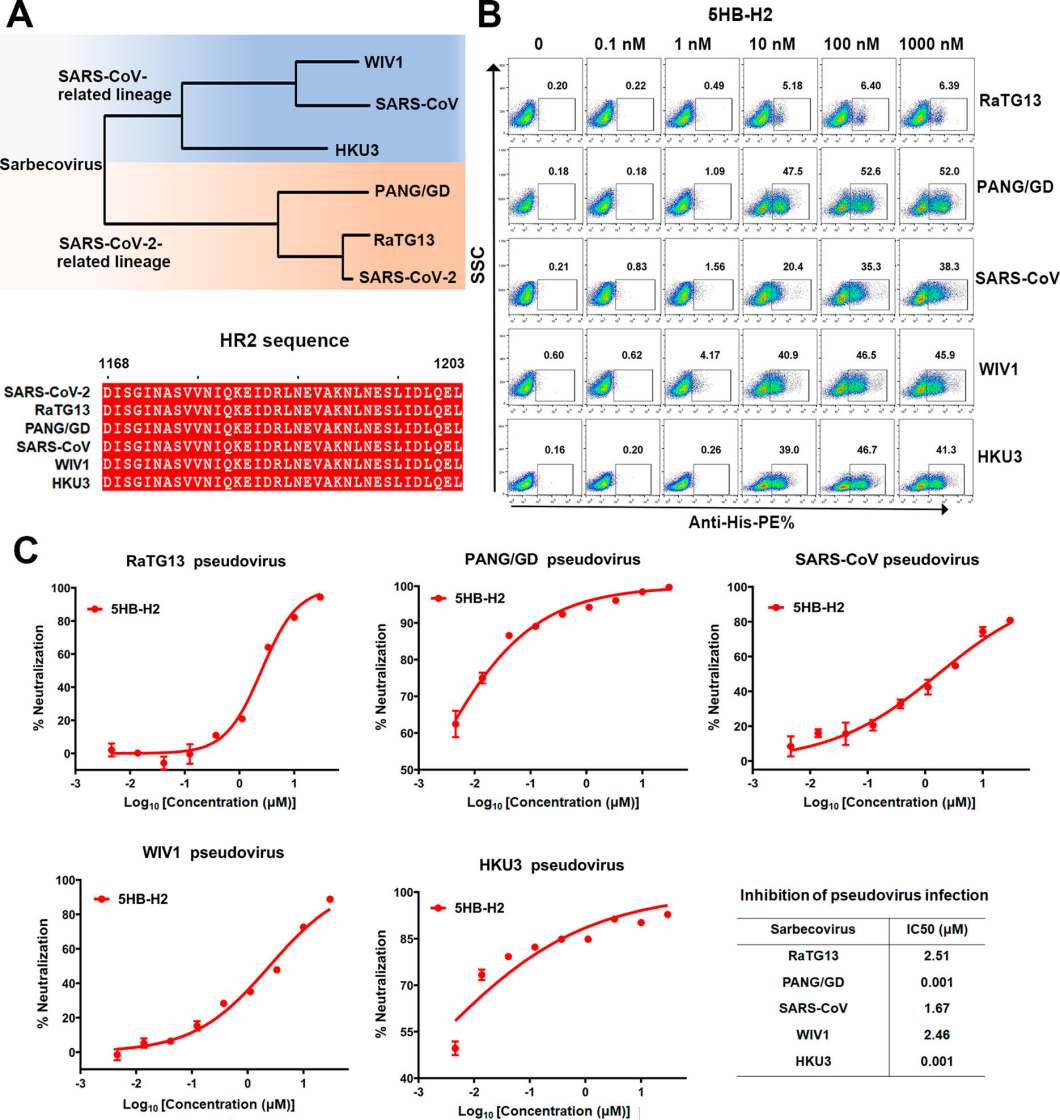
5HB-H2 broadly neutralizes sarbecoviruses. (A) Phylogenetic tree of the sarbecoviruses S protein constructed via maximum likelihood analysis of amino acid sequences retrieved from GenBank. The HR2 sequences involved in 5HB-H2 interactions were completely conserved in sarbecoviruses, including SARS-CoV-2, RaTG13, PANG/GD, SARS-CoV, WIV1 and HKU3. (B) Binding avidity of 5HB-H2 protein to the S proteins of sarbecoviruses detected by flow cytometry. The ratio of stained cells was shown. (C) 5HB-H2 shows a broad-spectrum inhibitory activity against a variety of sarbecoviruses. Pseudoviruses were pre-incubated with 5HB-H2 at the indicated concentration and then were used to infect 293T-hACE2 cells. Inhibition of viral entry was measured according to the reduction in luciferase activities. Data are expressed as means ± SD. Error bars indicate standard deviation of triplicates.

Within the sarbecovirus group, SARS-CoV-2 and SARS-CoV are representative members of two evolutionary lineages (the SARS-CoV-2 related lineage and SARS-CoV related lineage). In addition to these two highly transmissible and pathogenic viruses, several other sarbecoviruses, including RaTG13, PANG/GD, WIV1 and HKU3, have drawn substantial attention due to the zoonotic potentials and their genomic sequence homologies to either SARS-CoV-2 or SARS-CoV. To investigate the effect of 5HB-H2 in inhibiting the entry of these sarbecoviruses, we firstly compared their spike HR2 sequences. The alignment result revealed completely conserved HR2 amino acids (Figure 6(A)). We next evaluated the interaction of 5HB-H2 with individual sarbecoviral S by flow cytometry. Potent binding by 5HB-H2 was expectedly recorded for all the sarbecoviruses tested (Figure 6(B)). We noticed that the binding of 5HB-H2 with RaTG13 spike-expressing cells seemed to saturate at about 6% in our flow-cytometric study. This might result from the low expression level of RaTG13 S in 293T cells. Accordingly, we also observed low pseudovirus production efficiency with RaTG13 spike. The entry-inhibition capacity of 5HB-H2 against these sabercoviruses were then determined using the pseudovirus. The IC50s were calculated to be 2.51 μM for RaTG13, 1.67 μM for SARS-CoV, 2.46 μM for WIV1, and 0.001 μM for both PANG/GD and HKU3, respectively (Figure 6(C)). The results demonstrate that 5HB-H2 shows a broad-spectrum inhibitory activity against a variety of sarbecoviruses.

## Discussion

Despite global efforts, SARS-CoV-2 is still spreading worldwide and has caused irreparable harm to public health and social economy. Although vaccines and monoclonal antibody therapies are currently available, constant evolution of the virus has dramatically complicated the COVID-19 pandemic situations. Within viral S, RBD is the most prevalent target for neutralizing antibodies. Unfortunately, RBD is variable during virus evolution [39]. The circulating SARS-CoV-2 variants, including Alpha, Beta, Delta and Omicron, all bear RBD mutations that could compromise antibody efficacy [11,13,14,15]. In particular, it is worth noting that the newly-emergent Omicron variant possesses 15 substitutions in RBD [40]. Over 85% of the tested neutralizing antibodies targeting RBD are shown to be escaped [15]. In face of these urgent issues, potential therapeutics with cross-variant antiviral efficacy are urgently needed. One way to tackle the problem is to identify therapeutic agents, as exemplified by the decoy ACE2 mutants with increased RBD-binding capacity [41,42], to tolerate S mutations; and the other is to target the conserved regions in viral S, as represented by the HR1-targeting peptide inhibitors [28,29,34,43], for drug development. Unlike RBD that is prone to mutation during evolution, HR1 and HR2 of S2 are highly conserved. The fusion core composed of HR1 and HR2 is therefore an attractive drug target. By targeting HR1, peptides derived from HR2 have been shown in several studies to be able to inhibit viral entry of SARS-CoV-2 and the related coronaviruses [26,27,34]. Reciprocally by targeting HR2, recombinant 5HB proteins have been successfully designed, for HIV, MERS-CoV and very recently for SARS-CoV-2[32,33,44], to block the virus infection. In the current study, we similarly focus on the fusion core of SARS-CoV-2 and have designed the 5HB proteins with clear anti-SARS-CoV-2 efficacy.

The 5HB proteins expectedly possess broad neutralization capability. The optimal construct, 5HB-H2, not only inhibits infection of the prototype SARS-CoV-2 and the Omicron-included variants, but also blocks the entry of SARS-CoV-2 and SARS-CoV related sarbecoviruses. Via the structural study, we have shown that 5HB-H2 assembles into the proper five-helical structure and binds viral S via the canonical HR1/HR2 interactions. It is worth noting that the HR2 region recognized by 5HB-H2 in our structure is highly conserved, such that the amino acid sequences are 100% identical among SARS-CoV-2, SARS-CoV and other sarbecoviruses. Noted that HR1s of these viruses are observed to contain, though limited but not rare, sequence variations, the efficacy of HR1-targeting peptides might therefore be affected when dealing with different sarbecoviruses. In this sense, we believe that 5HB-H2 represents a good candidate for future development towards a pan-sarbecovirus drug. In light of the possible zoonotic nature of SARS-CoV-2 in a way that it evolves to infect humans via cross-species transmission [45], the threats from other sarbecoviruses should never be ignored. For example, both RaTG13- and PANG/GD-S are able to bind human ACE2 [46,47], suggesting the capacity of these viruses for potential cross-species transmission.

In comparison to the HR1-targeting peptides, an interesting feature of the 5HB protein is its capacity to bind viral S in the pre-fusion form. On virion surface, pre-S trimer is the dominate species prior to ACE2 binding. The capability of binding to pre-fusion S would, in our opinion, confer the 5HB protein a longer function-window to exert inhibition, superior to the HR2 derived peptides that are suggested to take action only in the fusion intermediate. Such pre-bound characteristic of 5HB makes the protein more mimic the S2-targeting antibody, such as S2P6[30]. Nevertheless, the antigen/antibody interactions are commonly compromised by acidic pH. At endosomal pH, the spike binding ability of S2P6 is shown to be decreased by about sixfold in comparison to that at serological pH [30]. In some cases as exemplified by the CR3022 antibody, such low-pH induced affinity decrease could be up to 1000-fold [48]. In contrast, we find that 5HB binds S in an acidification-insensitive manner. As a matter of fact, the binding affinity of 5HB-H2 to HR2P seems to be slightly increased along pH reduction. We therefore believe that, with this unique feature, 5HB-H2 represents a promising inhibitor capable of functioning when fusion occurs either at the cell surface or within the endosomes.

Despite of the advantages such as broad neutralization, readiness to bind pre-S and intimate binding at both serological- and endosomal-pH, the inhibition efficacy of 5HB seems not very “eye-catching.” In our evaluations, 5HB-H2 shows sub-micromolar IC50s (0.63 μM in the cell–cell fusion assay and 0.59 μM in the pseudovirus entry inhibition assay). These values coincide well with previous 5HB studies in other viruses. For instance, an engineered 5HB protein targeting MERS-CoV inhibits MERS-CoV pseudovirus entry with an IC50 of about 1 μM [33]. A 5-Helix derived from the fusion core of Newcastle disease virus (NDV) shows an IC50 of 2.67 μM against an NDV avirulent strain [49]. In light of multiple studies reporting the improvement of virus inhibition activities for therapeutic agents via structure-guided mutation trail [41,50] or by bivalency with Fc [51], 5HB-H2 might be modified similarly in the future to increase its entry-inhibition capacity. It is also notable that soluble 5HB proteins are also proposed to act as potential vaccine candidates [32,49]. It would therefore be interesting to evaluate the antigenicity and immune protection of 5HB-H2 against SARS-CoV-2 in the future.

## Materials and methods

### Gene cloning, protein expression, and purification

Following the construct of 5HB of MERS-CoV [33], we designed the SARS-CoV-2 5HB by arranging three copies of HR1 and two copies of HR2 alternatively in a tandem array, linked by SGGRGG or GGSGGS. The longest construct, namely 5HB-H1, spans residues 918-974 of HR1 and residues 1164-1203 of HR2. The other three constructs are truncated ones with truncation of several HR1 helical turns and the corresponding HR2 loops either on one side or from both sides, including 5HB-H2 with amino acids of HR1 E918-L966 and HR2 D1168-L1203, 5HB-H3 with amino acids of HR1 A924-L966 and HR2 D1168-I1198 and 5HB-H4 with amino acids of HR1 E918-Q954 and HR2 V1176-L1203. The genes for SARS-CoV-2 5HB constructs were synthesized in the pET-21a vector via the Nde I and Xho I restriction sites at Convenience Biology, with a 6×His tag added at the C-terminus to facilitate protein purification.

For protein expression, the pET-21a-SARS-CoV-2-S-5HB expression vectors were transformed into *E. Coli* strain BL21 (DE3) competent cells. Cells were cultured and induced by 0.2 mM isopropyl-β-D-thiogalactoside (IPTG) at 16°C, overnight. For protein purification, cells were initially collected, lysed by a high-pressure homogenizer, and clarified via centrifugation. The cell lysate was passed over 5 mL HisTrap (Qiagen) for affinity chromatography. Subsequently, soluble proteins were loaded on a Source 15Q column (GE Healthcare) for ion exchange and then a Superdex 200 Increase 10/300 GL column (GE Healthcare) for gel filtration. The UV absorbance curves were recorded at 215-nm since the lack of aromatic residues. The polyacrylamide gel electrophoresis was used to determine the purity of the final protein preparation.

To obtain a comparable molecular weight with our 5HB proteins, we fused the EK1 and HR2P peptides with an N-terminal Trx tag, and an N-terminal 6-His tag to facilitate purification, named Trx-EK1 and Trx-HR2 hereafter. The N-terminal His-tagged Trx protein alone was also designed for control. The above-designed coding sequences were amplified and subcloned into the pET-21a vector via the Nde I and Xho I restriction sites. The sequencing-verified plasmid was subsequently transformed into *E. Coli* strain BL21 (DE3) competent cells for protein expression. The purification method of Trx-EK1 and Trx-HR2 proteins is the same as the 5HB proteins.

### Crystallization

The homologous 5HB-H2 protein was mixed with HR2P at the molar ratio of 1:1.5 for 1 h on ice. The extra HR2P was excluded by gel filtration on a Superdex 200 Increase 10/300 GL column. Subsequently, the 5HB-H2/HR2P complex was concentrated to 2.5 mg/mL or 5 mg/mL for crystallization by using the commercial crystallization kits (Molecular Dimensions and Hampton Research). Diffractable crystals were obtained by mixing 1 μL protein with 1 μL reservoir solution consisting of 0.1 M Citric acid (pH 3.5) and 14% w/v Polyethylene glycol 1000 and then equilibrated against 75 μL reservoir solution at 16°C via vapor-diffusion sitting-drop method.

### Data collection and structure determination

For data collection, crystals were flash-cooled in liquid nitrogen after a brief soaking in a reservoir solution supplemented with 20% (v/v) glycerol. X-ray diffraction data were collected at Shanghai Synchrotron Radiation Facility (SSRF) beamline BL19U [52]. All data were carried out with HKL2000 [53] for indexing, integration, and scaling. Structures were determined by the molecular replacement method using Phaser [54] in the CCP4 suite [55]. Coordinates of SARS-CoV-2 S-6HB (PDB code: 6LXT) were used as the search model. Initial restrained rigid-body refinement was conducted using REFMAC5 [56], followed by manual rebuilding and adjustment in COOT [53]. Further refinement was processed using Phenix [57]. The stereochemical qualities of the final models were assessed through the programme PROCHECK [58]. The final data processing and structure refinement statistics are listed in Table. S1. All structural figures were generated with PyMOL (http://www.pymol.org).

### Bio-layer interferometry (BLI) assay

BLI assay was performed on an Octet Red 96 System (Forté Bio) in serials of assay buffer, respectively, containing 20 mM HEPES (pH 7.5) or 20 mM MES (pH 6.0) or 20 mM MES (pH 5.5) or 20 mM Glycine (pH 5.0) or 20 mM Glycine (pH 4.5) with 150 mM NaCl, 0.05% (v/v) Tween 20 at 298 K. The measurements were carried out by using SA biosensors. The 5HB proteins were biotinylated with the Biotinylation Kit (BMD Lab service, G-MM-IGT) according to the manufacturer's instructions, and immobilized on the biosensor surface. Serial dilutions of the HR2P from 7.5 nM to 120 nM were employed. Data analysis was conducted using ForteBio Data Analysis 12.0 programme with a 1:1 binding mode. For each binding experiment on a certain pH, three independent kinetic assays were conducted, and the calculated kinetic parameters were summarized.

### Isothermal titration calorimetry (ITC) assay

ITC measurements were performed on a Microcal PEAQ-ITC instrument (Malvern) at 298 K, with a reference power of 10 cal/s and a stirring speed of 750 rpm. Proteins and peptides were initially prepared in ITC buffer, containing 20 mM HEPES (pH 7.5) and 150 mM NaCl. Each titration typically involves 20 injections of 2 μL peptide with 4 s durations and 150 s intervals. The data fitting and analyses were performed using the MicroCal PEAQ-ITC Analysis Software.

### Enzyme-linked immunosorbent assay (ELISA)

We synthesized a peptide HR2P representative of SARS-CoV-2 HR2 with purity above 95% (Synpeptide Co., Ltd), for determining the interaction between 5HB proteins and HR2. The sequence of synthesized HR2P peptide is DISGINASVVNIQKEIDRLNEVAKNLNESLIDLQEL. 96-well microtiter plates were initially coated with HR2P at 200 ng/well in 0.05 M carbonate–bicarbonate buffer (pH 9.6) at 4°C overnight. The wells were then blocked with 5% skimmed milk solved in PBS, for 1 h at room temperature. After blocking, 2 μM 5HB-H1, 5HB-H2, 5HB-H3, 5HB-H4, and Trx protein were added to the wells and incubated for 1.5 h, followed by the addition of rabbit anti-His-HRP (Merck Millipore) and incubation for another 1 h. Trx protein was used as a negative control. In each step, the wells were fully washed with PBST. The reaction was stopped by the addition of 2 M HCl. The emission OD450 was monitored using a microplate reader (Thermo).

### Inhibition of SARS-CoV-2 S-mediated cell–cell fusion

The inhibitory activity of proteins or peptides on SARS-CoV-2 S-mediated cell–cell fusion was detected as previously described [34]. Briefly, 293T cells were transfected with plasmid pCAGGS-Kozak-EGFP (named 293T/EGFP). 293T cells were co-transfected with plasmid pCAGGS-Kozak-SARS-CoV-2 S and pCAGGS-Kozak-EGFP as the effector cells (named 293T/S/EGFP). ACE2 (GenBank: BAB40370.1) stable-expressed 293T cells (hereafter named 293T-hACE2) were prepared as our previous research and used as the target cells [41]. 2.5 × 10^4^ effector cells (293T/S/EGFP cells) were firstly mixed with indicated proteins (5HB-H1, 5HB-H2, 5HB-H3, 5HB-H4, Trx-HR2, and Trx-EK1) or peptides (HR2P and EK1) for 20 min at 37°C. Next, the mixtures were co-cultured with 5 × 10^4^ target cells for cell–cell fusion in DMEM with 10% fetal bovine serum (FBS). After incubation for 4 h, three fields were randomly selected and pictured in each well by fluorescence microscope (Nikon). ImageJ software (version 1.46) was used to quantify the area of fused and unfused cells. The (P-E)/(P-N) × 100% formula was used to calculate the inhibition activity of cell–cell fusion, as previously described [29]. “P,” “N” and “E,” represents the green areas of cell–cell fusion in the positive group without any inhibitor, negative group (293T/S/EGFP cells with PBS), and experimental groups (293T/S/EGFP cells were co-cultured with 293T-ACE2 cells in the presence of inhibitors), respectively. The IC50 was calculated using the GraphPad Prism 8 software. Samples were tested in triplicate, and experiments were repeated twice.

### Pseudovirus packing and inhibition of pseudoviral infections

Pseudotyped viruses were packed as our previously described [41]. Briefly, the S genes of sarbecovirus including SARS-CoV-2, PANG/GD, RaTG13, SARS-CoV, WIV1, and HKU3 (GenBank: MN908947.3, QLR06867.1, QHR63300.2, AY278554.2, AGZ48828.1, and QND76034.1, respectively), were individually synthesized in pCAGGS vector via the EcoR I and Bgl II restriction sites at Convenience Biology. The respective S plasmid and an Env-defective luciferase-expressing HIV-1 (pNL4-3.luc.RE) plasmid were co-transferred into 293T cells using Lipo8000 (Beyotime). After transfection for 48 h, supernatants were concentrated to gather pseudovirus and stored at −80°C for use. Pseudoviruses for SARS-CoV-2 variants (B.1.351, B.1.617.1, B.1.617.2, B.1.1.529) were purchased from Genomeditech company (#GM-0220PV32, #GM-0220PV44, #GM-0220PV45 and #GM-0220PV84, respectively).

Inhibition of pseudovirus infection assay was conducted as followed. First, 293T-hACE2 cells (1 × 10^4^ cells/well in 96-well plates) were cultured for adherence before infection. Next, pseudoviruses were pre-incubated with respective 5HB proteins (5HB-H1, 5HB-H2, 5HB-H3, respectively) at the indicated concentrations for 1 h at 37°C. Then the mixtures were added to 293T-hACE2 cells. After infection for 24 h, cells were re-fed with fresh DMEM with 10% FBS for an additional 48 h. Finally, the luciferase activity was detected using the One-LumiTM II Firefly Luciferase Assay Kit according to the manufacturer’s instructions (Beyotime).

### Flow cytometry assay

The binding of 5HB proteins with sarbecoviruses S was detected by flow cytometry. Briefly, 293T cells were transfected with plasmids encoding the wild-type S of SARS-CoV-2, SARS-CoV, RaTG13, PANG/GD, HKU3, and WIV1, respectively. Plasmid of SARS-CoV-2 S with mutations (K986P, V987P, S383C, and D985C) was also transfected into 293T cells to express SARS-CoV-2 S with pre-fusion conformation [37]. After transfection for 48 h, 5 × 10^5^ cells were pre-incubated with tested proteins, including 5HB-H1, 5HB-H2, 5HB-H3 or 5HB-H4 with C-His tag, Trx-HR2, or Trx-EK1 with N-His tag, HR2P or EK1 peptides with C-His tag at a serially diluted concentration for 1.5 h, respectively. Unbound molecules were washed out by centrifuging and resuspending with PBS. Next, cells were stained with anti-His-PE (Miltenyi Biotec, #130-120-718) at 4°C for 10 min. Finally, cells were washed with PBS to remove the non-specific binding antibodies and were monitored by flow cytometry (ACEA NovoCyte). All data were analyzed with Flowjo 10 software.

### Circular dichroism spectroscopic analysis

Circular dichroism spectra (200–260 nm at 25°C) were collected on a CIRCULAR DICHRIOSM SPECTROMETER MODEL 400 (AVIV) to evaluate the secondary structure of the SARS-CoV-2 5HB proteins. Indicated proteins were adjusted to a final concentration at 0.5 mg/ml in PBS. Data was collected by taking data points every 1-nm with a bandwidth of 1-nm.

## Supplementary Material

Supplemental Material

## Data Availability

The coordinates and the related structural factors for 5HB-H2/HR2P complex have been deposited into the Protein Data Bank with a PDB code of 7Y9N.
