# Supplementary material for: An engineered 5-helix bundle derived from SARS-CoV-2 S2 pre-binds sarbecoviral spike at both serological- and endosomal-pH to inhibit virus entry
Source: Emerg Microbes Infect. 2022 Aug 5;11(1):1920–35. doi: 10.1080/22221751.2022.2095308 (PMC9359175; doi:10.1080/22221751.2022.2095308)
Supplement: Supplemental Material [file TEMI_A_2095308_SM3862.docx]

Supplementary Materials for

An engineered 5-helix bundle derived from SARS-CoV-2 S2 pre-binds sarbecoviral spike at both serological- and endosomal-pH to inhibit virus entry

Xi Lin^1†^, Liyan Guo^1†^, Sheng Lin^1^, Zimin Chen^1^, Fanli Yang^1^, Jing Yang^1^, Lingling Wang^1^, Ao Wen^1^, Yanping Duan^1^, Xindan Zhang^1^, Yushan Dai^1^, Keqing Yin^1^, Xin Yuan^1^, Chongzhang Yu^1^, Bin He^1^, Yu Cao^1,2^, Haohao Dong^3^, Jian Li^4^, Qi Zhao^5^, Guangwen Lu^1*^

Correspondence to: lugw@scu.edu.cn

**This file includes:**

Figures. S1 to S4

Tables. S1 to S2

**Figure. S1**


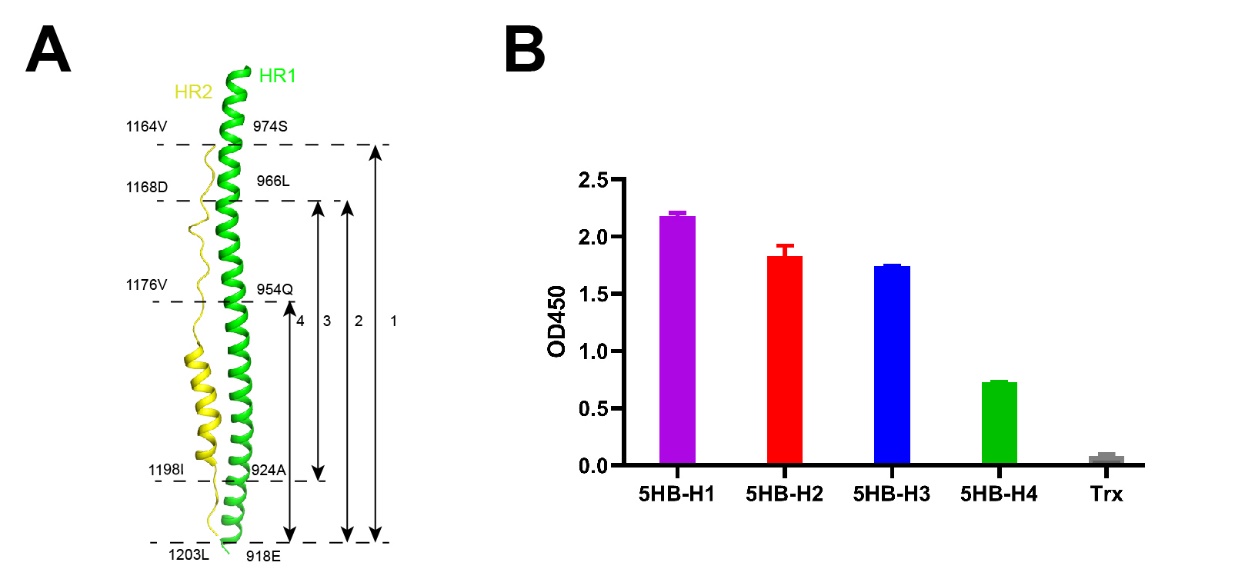


**Fig. S1 The schematic diagram of the designed 5HB proteins and its binding performance with HR2P. (A)** A cartoon view of the designed 5HB proteins. The 1, 2, 3 and 4 numbers individually indicate the residue span of HR1 and HR2 for 5HB-H1, 5HB-H2, 5HB-H3 and 5HB-H4, respectively. HR1 is in green and HR2 is in yellow. **(B)** ELISA-binding profiles between 5HB proteins and HR2P. Trx protein was used as a negative control.

**Figure. S2**


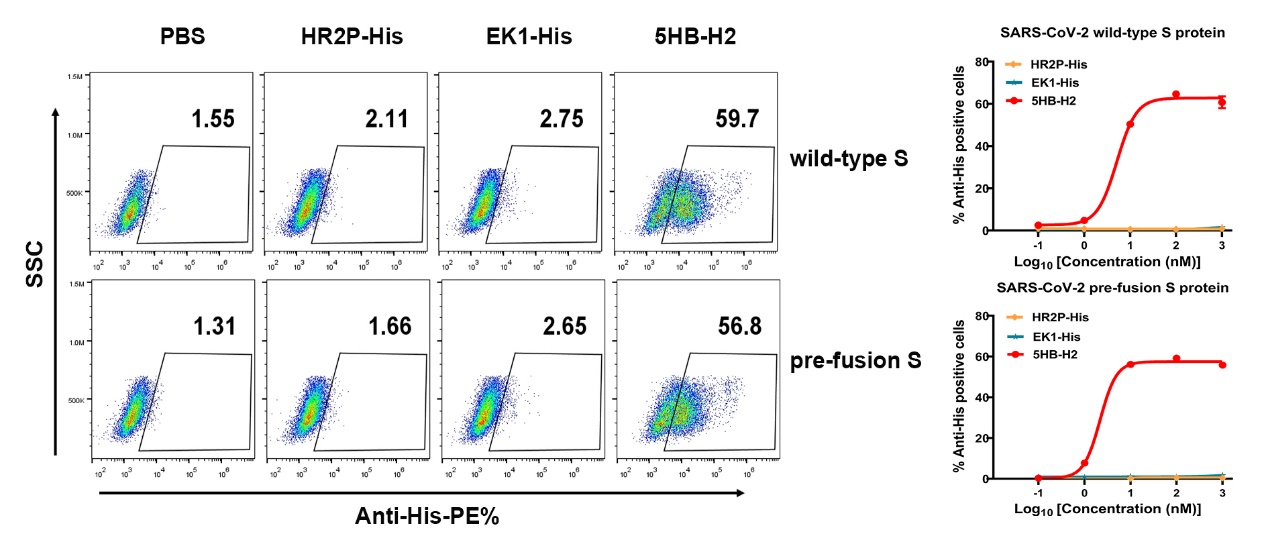


**Fig. S2 HR1-targeting peptides (HR2P and EK1) hardly bound to the wild-type or the pre-fusion S on the 293T cell surface.** Flow cytometric analysis of the effect of HR2P-His or EK1-His peptides on the wild-type or pre-fusion S binding (left). The 5HB-H2 protein was used as a positive control. The ratio of stained cells was shown. Mean fluorescence intensity was measured for HR2P-His or EK1-His binding to the wild-type or pre-fusion S expressed on the surface of 293T cells, with a 10-fold dilution for protein or peptides (right).

**Figure. S3**


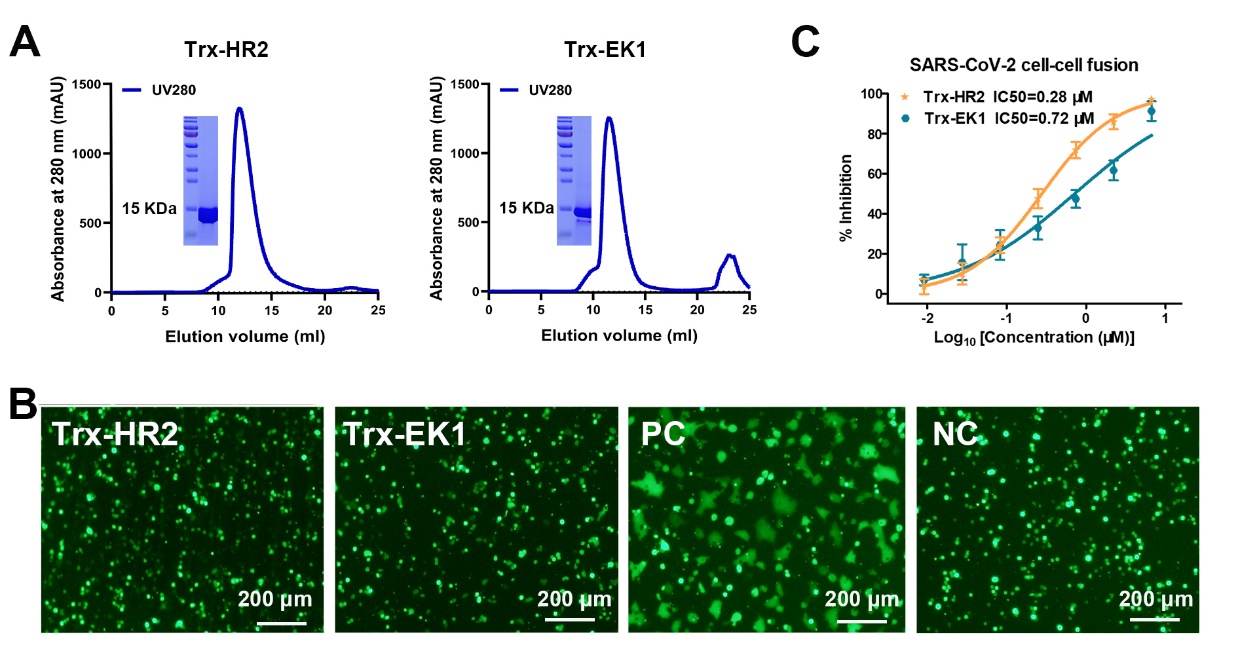


**Fig. S3 The entry-inhibition activity of Trx-HR2 and Trx-EK1 proteins. (A)** The solution behaviors of Trx-HR2 and Trx-EK1 proteins on a Superdex 75 10/300 column. **(B)** Representative images of SARS-CoV-2 S-mediated cell-cell fusion in the presence of Trx-HR2 or Trx-EK1 at 10 μM, with a scale bar of 200 μm. **(C)** Inhibitory activity of Trx-HR2 and Trx-EK1 proteins on SARS-CoV-2 S-mediated cell-cell fusion.

**Figure. S4**


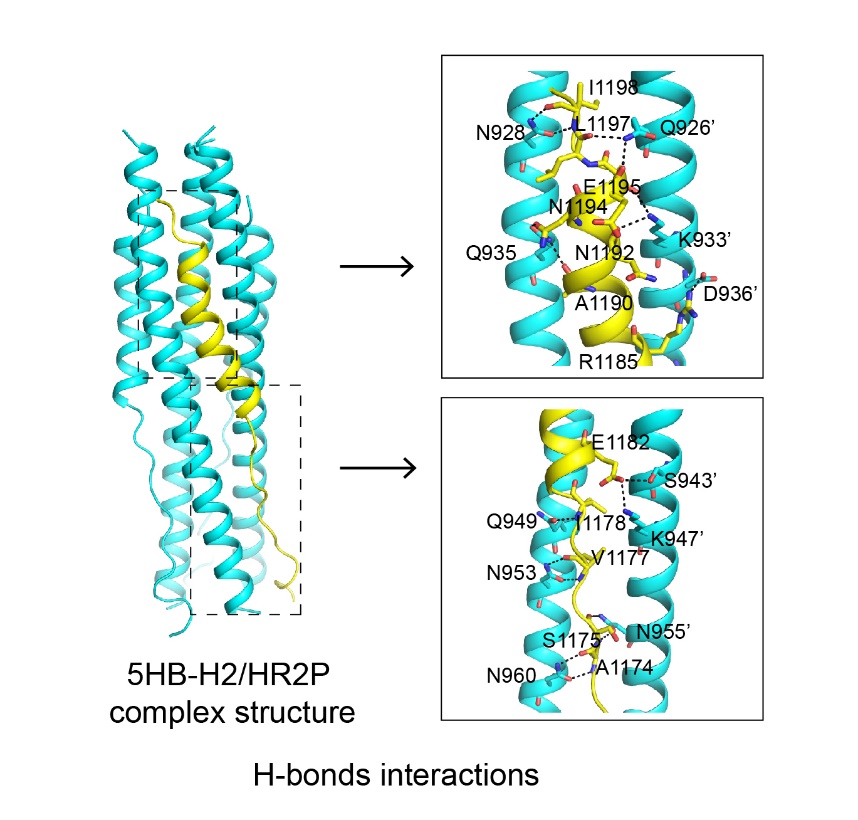


**Fig. S4** **The hydrogen-bond interaction details of 5HB-H2 and HR2P peptide.** Amino acids involved in the H-bond interactions are indicated and shown in sticks. 5HB-H2 are colored in cyan and HR2P in yellow. The distance cut-off is 3.1 Å.

**Table S1. Data collection and structure refinement statistics**

|  | SARS-CoV-2 S 5HB/HR2P complex structure |
| --- | --- |
| **Data collection** |  |
| Space group | C2 |
| Cell dimensions |  |
| *a*, *b*, *c* (Å) | 69.98, 40.50, 88.01 |
| α, β, γ (°) | 90.00, 105.31, 90.00 |
| Wavelength (Å) | 0.97852 |
| Resolution (Å) | 50-1.90 (1.97-1.90) |
| *R*_merge_ | 0.102 (0.640) |
| *I* / sig*I* | 26.444 (3.111) |
| Completeness (%) | 99.6 (99.5) |
| Redundancy | 6.1 (6.3) |
|  |  |
| **Refinement** |  |
| Resolution (Å) | 25.248-1.885 |
| No. reflections | 19221 |
| *R*_work_ / *R*_free_ | 0.1999/0.2124 |
| No. atoms | 1986 |
| Protein | 1849 |
| Water | 137 |
| *B*-factors |  |
| Protein | 40.613 |
| Water | 37.661 |
| R.m.s. deviations |  |
| Bond lengths (Å) | 0.006 |
| Bond angles (°) | 0.705 |
| Ramachandran plot (%)  Favored region  Allowed region  Outlier region | 100.00  0  0 |

In each case, a single crystal was used to collect the data.

Values in parentheses are for the highest-resolution shell.

**Table S2. Residues in 5HB-H2 interfacing with HR2P involved in the H-bonds interactions.**

| **HR2P** | | **5HB-H2** | | **Distance (Å)** |
| --- | --- | --- | --- | --- |
| Residues | Atoms | Residues | Atoms |  |
| 1174 (ALA) | N [ N] | ^b^960 (ASN) | OD1 [O] | 2.67 |
| 1174 (ALA) | O [ O] | ^b^960 (ASN) | ND2 [N] | 3.03 |
| 1175 (SER) | N [ N] | ^a^955 (ASN) | OD1 [O] | 2.85 |
| 1175 (SER) | O [ O] | ^a^955 (ASN) | ND2 [N] | 2.98 |
| 1177 (VAL) | N [ N] | ^b^953 (ASN) | OD1 [O] | 2.79 |
| 1177 (VAL) | O [ O] | ^b^953 (ASN) | ND2 [N] | 2.95 |
| 1179 (ILE) | N [ N] | ^b^949 (GLN) | OE1 [O] | 2.81 |
| 1182 (GLU) | OE1[ O] | ^a^943 (SER) | OG [O] | 2.91 |
| 1182 (GLU) | OE1[ O] | ^a^947 (LYS) | NZ [N] | 3.04 |
| 1185 (ARG) | NH2[ N] | ^a^936 (ASP) | OD2 [O] | 2.87 |
| 1190 (ALA) | O [ O] | ^b^935 (GLN) | NE2 [N] | 2.68 |
| 1192 (ASN) | O [ O] | ^a^933 (LYS) | NZ [N] | 3.02 |
| 1194 (ASN) | OD1[ O] | ^b^935 (GLN) | NE2 [N] | 2.95 |
| 1195 (GLU) | O [ O] | ^a^926 (GLN) | NE2 [N] | 2.82 |
| 1196 (SER) | OG [ O] | ^a^933 (LYS) | NZ [N] | 3.01 |
| 1197 (LEU) | O [ O] | ^a^926 (GLN) | NE2 [N] | 2.88 |
| 1198 (ILE) | N [ N] | ^b^928 (ASN) | OD1 [O] | 2.94 |
| 1198 (ILE) | O [ O] | ^b^928 (ASN) | ND2 [N] | 2.69 |

a, one of central HR1 helix of 5HB-H2;

b, another central HR1 helix of 5HB-H2;

The distance cut-off is 3.1 Å.
